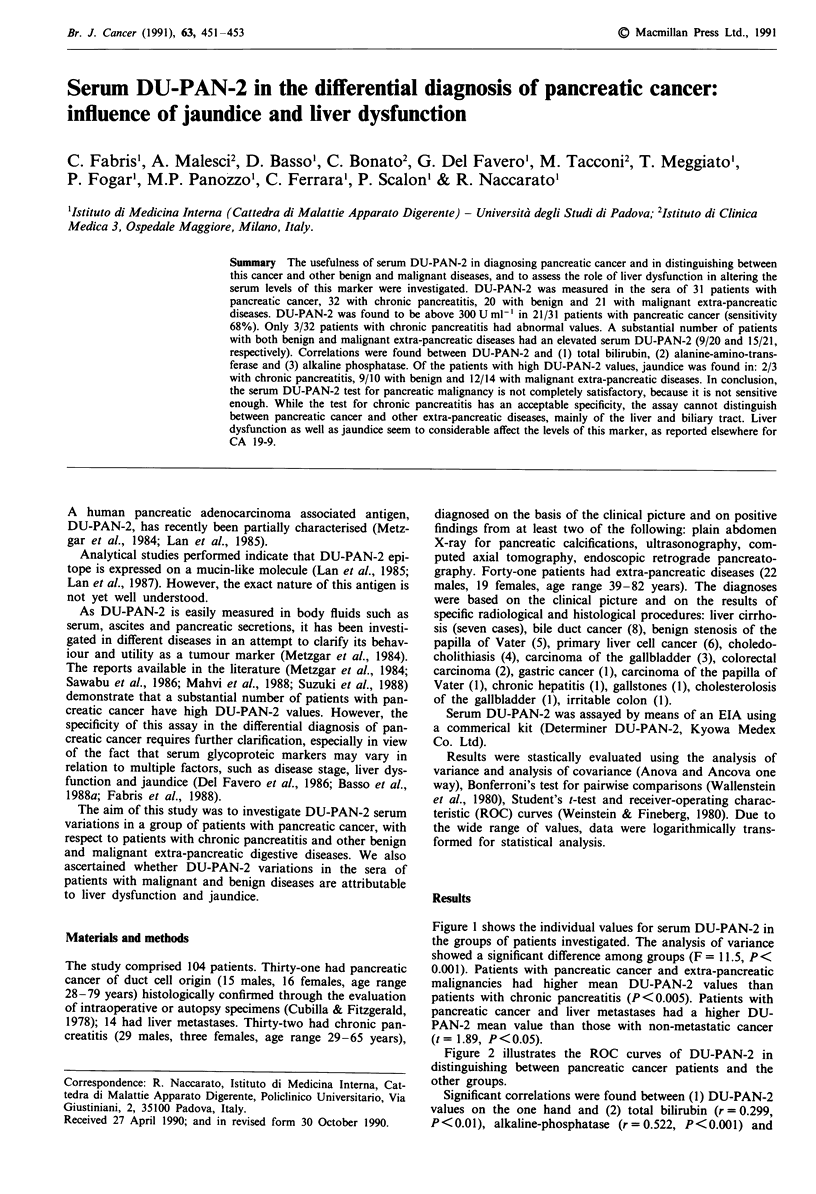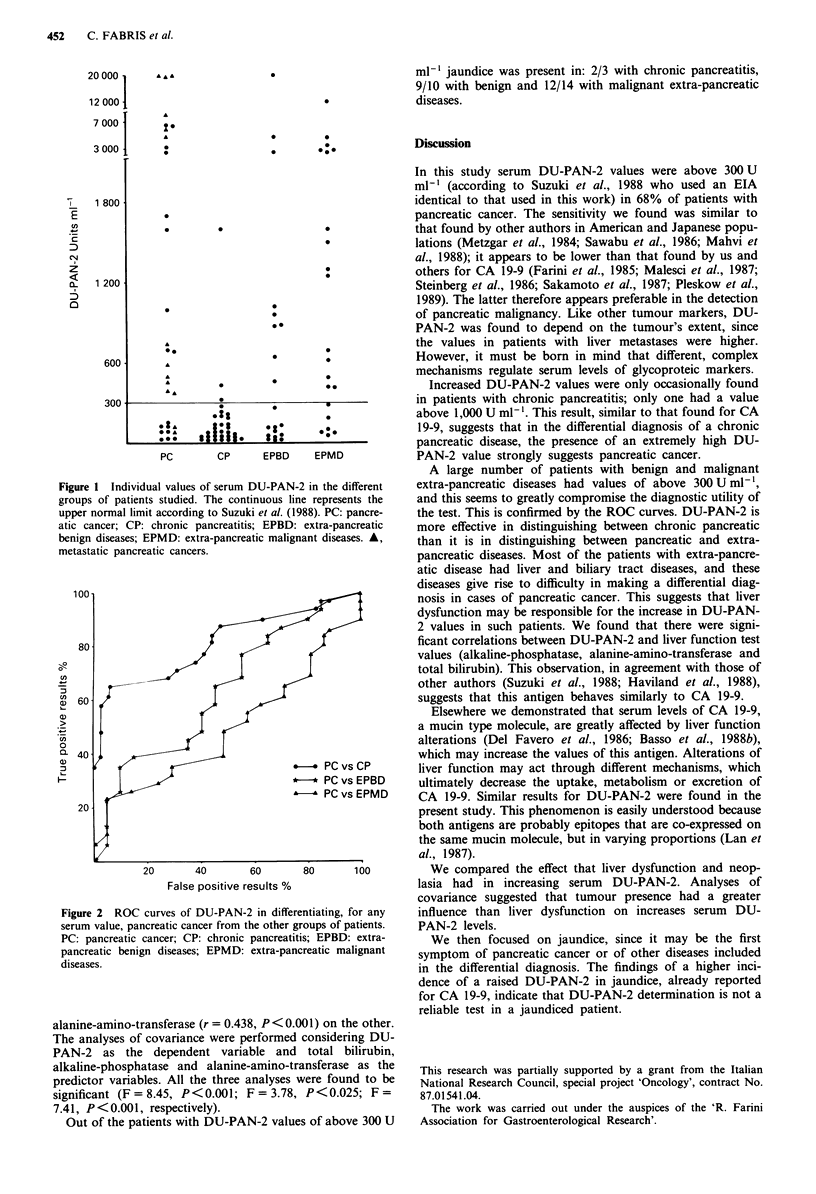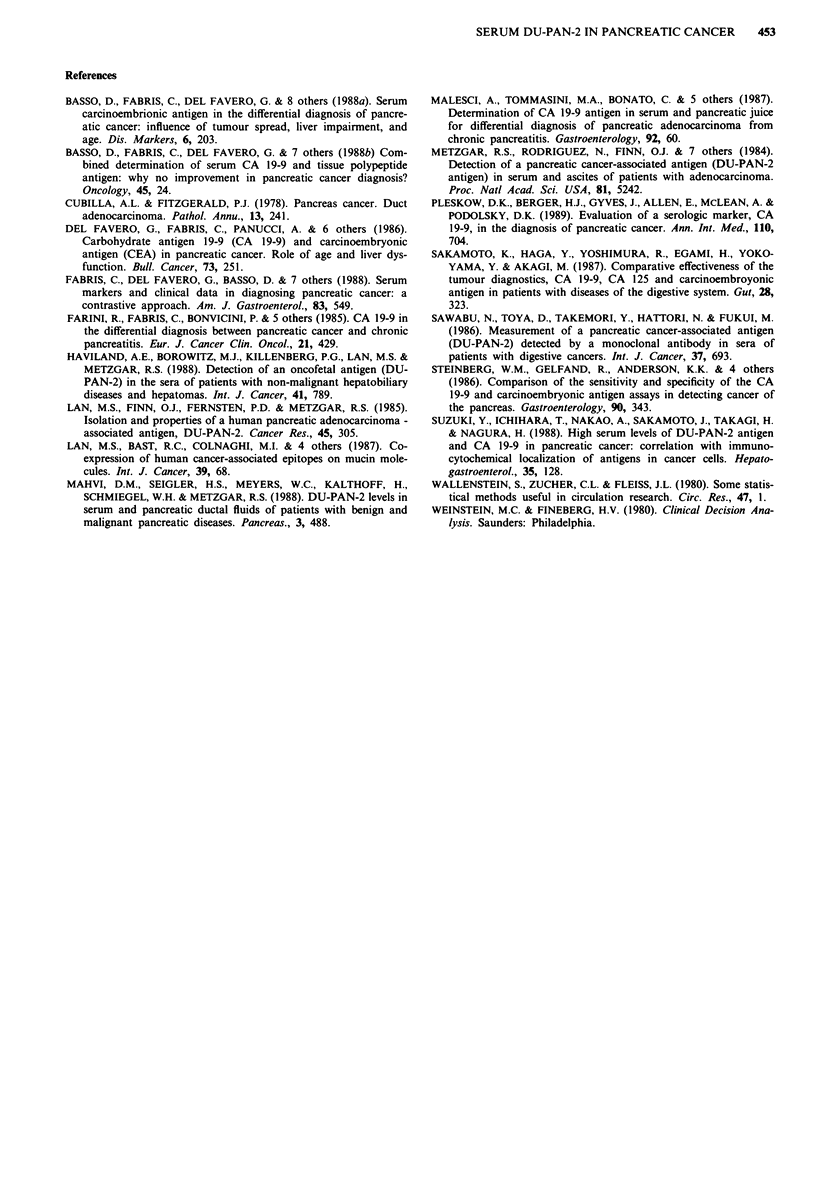# Serum DU-PAN-2 in the differential diagnosis of pancreatic cancer: influence of jaundice and liver dysfunction.

**DOI:** 10.1038/bjc.1991.104

**Published:** 1991-03

**Authors:** C. Fabris, A. Malesci, D. Basso, C. Bonato, G. Del Favero, M. Tacconi, T. Meggiato, P. Fogar, M. P. Panozzo, C. Ferrara

**Affiliations:** Istituto di Medicina Interna (Cattedra di Malattie Apparato Digerente), Università deglì Studi di Padova, Italy.

## Abstract

The usefulness of serum DU-PAN-2 in diagnosing pancreatic cancer and in distinguishing between this cancer and other benign and malignant diseases, and to assess the role of liver dysfunction in altering the serum levels of this marker were investigated. DU-PAN-2 was measured in the sera of 31 patients with pancreatic cancer, 32 with chronic pancreatitis, 20 with benign and 21 with malignant extra-pancreatic diseases. DU-PAN-2 was found to be above 300 U ml-1 in 21/31 patients with pancreatic cancer (sensitivity 68%). Only 3/32 patients with chronic pancreatitis had abnormal values. A substantial number of patients with both benign and malignant extra-pancreatic diseases had an elevated serum DU-PAN-2 (9/20 and 15/21, respectively). Correlations were found between DU-PAN-2 and (1) total bilirubin, (2) alanine-amino-transferase and (3) alkaline phosphatase. Of the patients with high DU-PAN-2 values, jaundice was found in: 2/3 with chronic pancreatitis, 9/10 with benign and 12/14 with malignant extra-pancreatic diseases. In conclusion, the serum DU-PAN-2 test for pancreatic malignancy is not completely satisfactory, because it is not sensitive enough. While the test for chronic pancreatitis has an acceptable specificity, the assay cannot distinguish between pancreatic cancer and other extra-pancreatic diseases, mainly of the liver and biliary tract. Liver dysfunction as well as jaundice seem to considerable affect the levels of this marker, as reported elsewhere for CA 19-9.


 © Macmillan Press Ltd., 1991

### Serum DU-PAN-2 in the differential diagnosis of pancreatic cancer: influence of jaundice and liver dysfunction

C. Fabris[1], A. Malesci[2], D. Basso[1], C. Bonato[2], G. Del Favero[1], M. Tacconi[2], T. Meggiato[1], P. Fogar[1], M.P. Panozzo[1], C. Ferrara[1], P. Scalon[1] & R. Naccarato[1]

[1]*Istituto di Medicina Interna (Cattedra di Malattie Apparato Digerente) – Università degli Studi di Padova;* [2]*Istituto di Clinica Medica 3, Ospedale Maggiore, Milano, Italy.*

**Summary** The usefulness of serum DU-PAN-2 in diagnosing pancreatic cancer and in distinguishing between this cancer and other benign and malignant diseases, and to assess the role of liver dysfunction in altering the serum levels of this marker were investigated. DU-PAN-2 was measured in the sera of 31 patients with pancreatic cancer, 32 with chronic pancreatitis, 20 with benign and 21 with malignant extra-pancreatic diseases. DU-PAN-2 was found to be above 300 U ml$^{-1}$ in 21/31 patients with pancreatic cancer (sensitivity 68%). Only 3/32 patients with chronic pancreatitis had abnormal values. A substantial number of patients with both benign and malignant extra-pancreatic diseases had an elevated serum DU-PAN-2 (9/20 and 15/21, respectively). Correlations were found between DU-PAN-2 and (1) total bilirubin, (2) alanine-amino-transferase and (3) alkaline phosphatase. Of the patients with high DU-PAN-2 values, jaundice was found in: 2/3 with chronic pancreatitis, 9/10 with benign and 12/14 with malignant extra-pancreatic diseases. In conclusion, the serum DU-PAN-2 test for pancreatic malignancy is not completely satisfactory, because it is not sensitive enough. While the test for chronic pancreatitis has an acceptable specificity, the assay cannot distinguish between pancreatic cancer and other extra-pancreatic diseases, mainly of the liver and biliary tract. Liver dysfunction as well as jaundice seem to considerable affect the levels of this marker, as reported elsewhere for CA 19-9.

A human pancreatic adenocarcinoma associated antigen, DU-PAN-2, has recently been partially characterised (Metzgar *et al.*, 1984; Lan *et al.*, 1985).

Analytical studies performed indicate that DU-PAN-2 epitope is expressed on a mucin-like molecule (Lan *et al.*, 1985; Lan *et al.*, 1987). However, the exact nature of this antigen is not yet well understood.

As DU-PAN-2 is easily measured in body fluids such as serum, ascites and pancreatic secretions, it has been investigated in different diseases in an attempt to clarify its behaviour and utility as a tumour marker (Metzgar *et al.*, 1984). The reports available in the literature (Metzgar *et al.*, 1984; Sawabu *et al.*, 1986; Mahvi *et al.*, 1988; Suzuki *et al.*, 1988) demonstrate that a substantial number of patients with pancreatic cancer have high DU-PAN-2 values. However, the specificity of this assay in the differential diagnosis of pancreatic cancer requires further clarification, especially in view of the fact that serum glycoproteic markers may vary in relation to multiple factors, such as disease stage, liver dysfunction and jaundice (Del Favero *et al.*, 1986; Basso *et al.*, 1988*a*; Fabris *et al.*, 1988).

The aim of this study was to investigate DU-PAN-2 serum variations in a group of patients with pancreatic cancer, with respect to patients with chronic pancreatitis and other benign and malignant extra-pancreatic digestive diseases. We also ascertained whether DU-PAN-2 variations in the sera of patients with malignant and benign diseases are attributable to liver dysfunction and jaundice.

### Materials and methods

The study comprised 104 patients. Thirty-one had pancreatic cancer of duct cell origin (15 males, 16 females, age range 28–79 years) histologically confirmed through the evaluation of intraoperative or autopsy specimens (Cubilla & Fitzgerald, 1978); 14 had liver metastases. Thirty-two had chronic pancreatitis (29 males, three females, age range 29–65 years), diagnosed on the basis of the clinical picture and on positive findings from at least two of the following: plain abdomen X-ray for pancreatic calcifications, ultrasonography, computed axial tomography, endoscopic retrograde pancreatography. Forty-one patients had extra-pancreatic diseases (22 males, 19 females, age range 39–82 years). The diagnoses were based on the clinical picture and on the results of specific radiological and histological procedures: liver cirrhosis (seven cases), bile duct cancer (8), benign stenosis of the papilla of Vater (5), primary liver cell cancer (6), choledocholithiasis (4), carcinoma of the gallbladder (3), colorectal carcinoma (2), gastric cancer (1), carcinoma of the papilla of Vater (1), chronic hepatitis (1), gallstones (1), cholesterolosis of the gallbladder (1), irritable colon (1).

Serum DU-PAN-2 was assayed by means of an EIA using a commerical kit (Determiner DU-PAN-2, Kyowa Medex Co. Ltd).

Results were stastically evaluated using the analysis of variance and analysis of covariance (Anova and Ancova one way), Bonferroni's test for pairwise comparisons (Wallenstein *et al.*, 1980), Student's *t*-test and receiver-operating characteristic (ROC) curves (Weinstein & Fineberg, 1980). Due to the wide range of values, data were logarithmically transformed for statistical analysis.

### Results

Figure 1 shows the individual values for serum DU-PAN-2 in the groups of patients investigated. The analysis of variance showed a significant difference among groups ($F = 11.5$, $P < 0.001$). Patients with pancreatic cancer and extra-pancreatic malignancies had higher mean DU-PAN-2 values than patients with chronic pancreatitis ($P < 0.005$). Patients with pancreatic cancer and liver metastases had a higher DU-PAN-2 mean value than those with non-metastatic cancer ($t = 1.89$, $P < 0.05$).

Figure 2 illustrates the ROC curves of DU-PAN-2 in distinguishing between pancreatic cancer patients and the other groups.

Significant correlations were found between (1) DU-PAN-2 values on the one hand and (2) total bilirubin ($r = 0.299$, $P < 0.01$), alkaline-phosphatase ($r = 0.522$, $P < 0.001$) and

Correspondence: R. Naccarato, Istituto di Medicina Interna, Cattedra di Malattie Apparato Digerente, Policlinico Universitario, Via Giustiniani, 2, 35100 Padova, Italy.
Received 27 April 1990; and in revised form 30 October 1990.

**Figure 1** Individual values of serum DU-PAN-2 in the different groups of patients studied. The continuous line represents the upper normal limit according to Suzuki *et al.* (1988). PC: pancreatic cancer; CP: chronic pancreatitis; EPBD: extra-pancreatic benign diseases; EPMD: extra-pancreatic malignant diseases. ▲, metastatic pancreatic cancers.

**Figure 2** ROC curves of DU-PAN-2 in differentiating, for any serum value, pancreatic cancer from the other groups of patients. PC: pancreatic cancer; CP: chronic pancreatitis; EPBD: extra-pancreatic benign diseases; EPMD: extra-pancreatic malignant diseases.

alanine-amino-transferase ($r = 0.438$, $P < 0.001$) on the other. The analyses of covariance were performed considering DU-PAN-2 as the dependent variable and total bilirubin, alkaline-phosphatase and alanine-amino-transferase as the predictor variables. All the three analyses were found to be significant ($F = 8.45$, $P < 0.001$; $F = 3.78$, $P < 0.025$; $F = 7.41$, $P < 0.001$, respectively).

Out of the patients with DU-PAN-2 values of above 300 U $ml^{-1}$ jaundice was present in: 2/3 with chronic pancreatitis, 9/10 with benign and 12/14 with malignant extra-pancreatic diseases.

### Discussion

In this study serum DU-PAN-2 values were above 300 U $ml^{-1}$ (according to Suzuki *et al.*, 1988 who used an EIA identical to that used in this work) in 68% of patients with pancreatic cancer. The sensitivity we found was similar to that found by other authors in American and Japanese populations (Metzgar *et al.*, 1984; Sawabu *et al.*, 1986; Mahvi *et al.*, 1988); it appears to be lower than that found by us and others for CA 19-9 (Farini *et al.*, 1985; Malesci *et al.*, 1987; Steinberg *et al.*, 1986; Sakamoto *et al.*, 1987; Pleskow *et al.*, 1989). The latter therefore appears preferable in the detection of pancreatic malignancy. Like other tumour markers, DU-PAN-2 was found to depend on the tumour's extent, since the values in patients with liver metastases were higher. However, it must be born in mind that different, complex mechanisms regulate serum levels of glycoproteic markers.

Increased DU-PAN-2 values were only occasionally found in patients with chronic pancreatitis; only one had a value above 1,000 U $ml^{-1}$. This result, similar to that found for CA 19-9, suggests that in the differential diagnosis of a chronic pancreatic disease, the presence of an extremely high DU-PAN-2 value strongly suggests pancreatic cancer.

A large number of patients with benign and malignant extra-pancreatic diseases had values of above 300 U $ml^{-1}$, and this seems to greatly compromise the diagnostic utility of the test. This is confirmed by the ROC curves. DU-PAN-2 is more effective in distinguishing between chronic pancreatic than it is in distinguishing between pancreatic and extra-pancreatic diseases. Most of the patients with extra-pancreatic disease had liver and biliary tract diseases, and these diseases give rise to difficulty in making a differential diagnosis in cases of pancreatic cancer. This suggests that liver dysfunction may be responsible for the increase in DU-PAN-2 values in such patients. We found that there were significant correlations between DU-PAN-2 and liver function test values (alkaline-phosphatase, alanine-amino-transferase and total bilirubin). This observation, in agreement with those of other authors (Suzuki *et al.*, 1988; Haviland *et al.*, 1988), suggests that this antigen behaves similarly to CA 19-9.

Elsewhere we demonstrated that serum levels of CA 19-9, a mucin type molecule, are greatly affected by liver function alterations (Del Favero *et al.*, 1986; Basso *et al.*, 1988*b*), which may increase the values of this antigen. Alterations of liver function may act through different mechanisms, which ultimately decrease the uptake, metabolism or excretion of CA 19-9. Similar results for DU-PAN-2 were found in the present study. This phenomenon is easily understood because both antigens are probably epitopes that are co-expressed on the same mucin molecule, but in varying proportions (Lan *et al.*, 1987).

We compared the effect that liver dysfunction and neoplasia had in increasing serum DU-PAN-2. Analyses of covariance suggested that tumour presence had a greater influence than liver dysfunction on increases serum DU-PAN-2 levels.

We then focused on jaundice, since it may be the first symptom of pancreatic cancer or of other diseases included in the differential diagnosis. The findings of a higher incidence of a raised DU-PAN-2 in jaundice, already reported for CA 19-9, indicate that DU-PAN-2 determination is not a reliable test in a jaundiced patient.

This research was partially supported by a grant from the Italian National Research Council, special project 'Oncology', contract No. 87.01541.04.

The work was carried out under the auspices of the 'R. Farini Association for Gastroenterological Research'.

### References

BASSO, D., FABRIS, C., DEL FAVERO, G. & 8 others (1988*a*). Serum carcinoembrionic antigen in the differential diagnosis of pancreatic cancer: influence of tumour spread, liver impairment, and age. *Dis. Markers*, **6**, 203.

BASSO, D., FABRIS, C., DEL FAVERO, G. & 7 others (1988*b*) Combined determination of serum CA 19-9 and tissue polypeptide antigen: why no improvement in pancreatic cancer diagnosis? *Oncology*, **45**, 24.

CUBILLA, A.L. & FITZGERALD, P.J. (1978). Pancreas cancer. Duct adenocarcinoma. *Pathol. Annu.*, **13**, 241.

DEL FAVERO, G., FABRIS, C., PANUCCI, A. & 6 others (1986). Carbohydrate antigen 19-9 (CA 19-9) and carcinoembryonic antigen (CEA) in pancreatic cancer. Role of age and liver dysfunction. *Bull. Cancer*, **73**, 251.

FABRIS, C., DEL FAVERO, G., BASSO, D. & 7 others (1988). Serum markers and clinical data in diagnosing pancreatic cancer: a contrastive approach. *Am. J. Gastroenterol.*, **83**, 549.

FARINI, R., FABRIS, C., BONVICINI, P. & 5 others (1985). CA 19-9 in the differential diagnosis between pancreatic cancer and chronic pancreatitis. *Eur. J. Cancer Clin. Oncol.*, **21**, 429.

HAVILAND, A.E., BOROWITZ, M.J., KILLENBERG, P.G., LAN, M.S. & METZGAR, R.S. (1988). Detection of an oncofetal antigen (DU-PAN-2) in the sera of patients with non-malignant hepatobiliary diseases and hepatomas. *Int. J. Cancer*, **41**, 789.

LAN, M.S., FINN, O.J., FERNSTEN, P.D. & METZGAR, R.S. (1985). Isolation and properties of a human pancreatic adenocarcinoma - associated antigen, DU-PAN-2. *Cancer Res.*, **45**, 305.

LAN, M.S., BAST, R.C., COLNAGHI, M.I. & 4 others (1987). Co-expression of human cancer-associated epitopes on mucin molecules. *Int. J. Cancer*, **39**, 68.

MAHVI, D.M., SEIGLER, H.S., MEYERS, W.C., KALTHOFF, H., SCHMIEGEL, W.H. & METZGAR, R.S. (1988). DU-PAN-2 levels in serum and pancreatic ductal fluids of patients with benign and malignant pancreatic diseases. *Pancreas.*, **3**, 488.

MALESCI, A., TOMMASINI, M.A., BONATO, C. & 5 others (1987). Determination of CA 19-9 antigen in serum and pancreatic juice for differential diagnosis of pancreatic adenocarcinoma from chronic pancreatitis. *Gastroenterology*, **92**, 60.

METZGAR, R.S., RODRIGUEZ, N., FINN, O.J. & 7 others (1984). Detection of a pancreatic cancer-associated antigen (DU-PAN-2 antigen) in serum and ascites of patients with adenocarcinoma. *Proc. Natl Acad. Sci. USA*, **81**, 5242.

PLESKOW, D.K., BERGER, H.J., GYVES, J., ALLEN, E., MCLEAN, A. & PODOLSKY, D.K. (1989). Evaluation of a serologic marker, CA 19-9, in the diagnosis of pancreatic cancer. *Ann. Int. Med.*, **110**, 704.

SAKAMOTO, K., HAGA, Y., YOSHIMURA, R., EGAMI, H., YOKOYAMA, Y. & AKAGI, M. (1987). Comparative effectiveness of the tumour diagnostics, CA 19-9, CA 125 and carcinoembroyonic antigen in patients with diseases of the digestive system. *Gut*, **28**, 323.

SAWABU, N., TOYA, D., TAKEMORI, Y., HATTORI, N. & FUKUI, M. (1986). Measurement of a pancreatic cancer-associated antigen (DU-PAN-2) detected by a monoclonal antibody in sera of patients with digestive cancers. *Int. J. Cancer*, **37**, 693.

STEINBERG, W.M., GELFAND, R., ANDERSON, K.K. & 4 others (1986). Comparison of the sensitivity and specificity of the CA 19-9 and carcinoembryonic antigen assays in detecting cancer of the pancreas. *Gastroenterology*, **90**, 343.

SUZUKI, Y., ICHIHARA, T., NAKAO, A., SAKAMOTO, J., TAKAGI, H. & NAGURA, H. (1988). High serum levels of DU-PAN-2 antigen and CA 19-9 in pancreatic cancer: correlation with immunocytochemical localization of antigens in cancer cells. *Hepatogastroenterol.*, **35**, 128.

WALLENSTEIN, S., ZUCHER, C.L. & FLEISS, J.L. (1980). Some statistical methods useful in circulation research. *Circ. Res.*, **47**, 1.

WEINSTEIN, M.C. & FINEBERG, H.V. (1980). *Clinical Decision Analysis*. Saunders: Philadelphia.